# First-in-Human Study to Evaluate the Safety and Efficacy of Anti-GDF15 Antibody AZD8853 in Patients with Advanced/Metastatic Solid Tumors

**DOI:** 10.1158/2767-9764.CRC-24-0565

**Published:** 2025-06-02

**Authors:** Benedito A. Carneiro, Olumide B. Gbolahan, Albiruni Abdul Abdul Razak, John F. Hilton, Arthur W. Lambert, John Hood, Michael Pluta, Veronique Bragulat, Elhan Sanai, Rakesh Kumar, Duncan I. Jodrell, Patricia M. LoRusso

**Affiliations:** 1 Division of Hematology/Oncology, Legorreta Cancer Center, Brown University, Providence, Rhode Island.; 2 Department of Hematology and Medical Oncology, Emory University, Atlanta, Georgia.; 3 Phase 1 Program, Department of Medical Oncology, Princess Margaret Cancer Centre, Toronto, Canada.; 4 Cancer Therapeutics Program, Ottawa Hospital Research Institute, University of Ottawa, Ottawa, Canada.; 5 Translational Medicine, AstraZeneca, Waltham, Massachusetts.; 6 Clinical Pharmacology, AstraZeneca, Cambridge, United Kingdom.; 7 Oncology Biometrics, AstraZeneca, Cambridge, United Kingdom.; 8 Oncology Early Development Clinical, AstraZeneca, Cambridge, United Kingdom.; 9 Oncology R&D, AstraZeneca, Gaithersburg, Maryland.; 10 University of Cambridge, Cambridge, United Kingdom.; 11 Yale University Cancer Center, New Haven, Connecticut.

## Abstract

**Purpose:**

Growth and differentiation factor 15 (GDF15) is overexpressed in multiple solid tumors and is thought to exert immunosuppressive effects in the tumor microenvironment. AZD8853 is an anti-GDF15 mAb.

**Patients and Methods:**

This first-in-human, phase I/IIa, open-label study (NCT05397171) assessed AZD8853 monotherapy in previously treated patients with advanced/metastatic microsatellite-stable colorectal cancer and urothelial carcinoma. The primary objective was safety including dose-limiting toxicities. Secondary objectives included efficacy, pharmacokinetics, and pharmacodynamics (PD), including free serum GDF15. Exploratory objectives included biomarkers of clinical activity and effects on cancer cachexia.

**Results:**

During dose escalation, 16 patients received AZD8853 300 mg (*n* = 3), 1,000 mg (*n* = 6), or 3,000 mg (*n* = 7) intravenously every 3 weeks; 15 patients had microsatellite-stable colorectal cancer; and one patient had urothelial carcinoma. By June 6, 2023, all patients had discontinued treatment. Thirteen (81.3%) patients had treatment-emergent adverse events (TEAE); most commonly diarrhea (31.3%), abdominal pain (31.3%), and decreased appetite (25%). Eight (50.0%) patients had grade ≥3 TEAEs, and six (37.5%) had serious TEAEs, none treatment related. There were no dose-limiting toxicities. The best response per RECIST v1.1 was stable disease in five (31.3%) patients and disease progression in 11 (68.8%) patients. AZD8853 showed linear pharmacokinetics with a half-life of 5 to 10 days, supporting every 3 weeks dosing. AZD8853 suppression of GDF15 was transient. There was no evidence of ctDNA clearance or dose-dependent changes in peripheral T cells. Changes in body weight showed no apparent trends.

**Conclusions:**

AZD8853 was well tolerated; however, no objective responses or PD effects were seen, and GDF15 suppression was not sustained. The study was terminated after dose escalation.

**Significance:**

GDF15 is upregulated in the tumor microenvironment and suppresses antitumor immune responses. In this first-in-human trial, the anti-GDF15 antibody AZD8853 was well tolerated in previously treated patients with advanced/metastatic solid tumors, but no objective responses or PD effects were seen and GDF15 suppression was not sustained.

## Introduction

The tumor microenvironment (TME) is a key driver of tumorigenesis and disease progression and represents an important target for anticancer immunotherapies. Although immune checkpoint inhibitors, including PD-(L)1 blockers, have become part of the standard of care for several malignancies, many patients have resistant or refractory disease (1). The immunosuppressive functions of the TME can limit the efficacy of anticancer therapies by inhibiting immune cell infiltration and activity (1, 2). Counteracting this immunosuppression offers a promising approach to anticancer therapy.

Growth and differentiation factor 15 (GDF15, also known as macrophage inhibitory cytokine 1) is a cell stress–responsive cytokine in the TGF-β superfamily (3, 4). It has one identified receptor, glial cell–derived neurotrophic factor family receptor α-like (GFRAL; ref. 5). GDF15 protects against tissue injury, acts as a metabolic and immune regulator, and may be a biomarker of obesity, cardiovascular diseases, and various cancers (3, 4, 6–10). GDF15 is overexpressed in several solid malignancies, including colorectal cancer, non–small cell lung cancer (NSCLC), urothelial carcinoma, and renal cell carcinoma (4, 6, 8, 9, 11–13). Elevated GDF15 expression is associated with poor prognosis and survival in multiple solid malignancies (8, 9, 11, 13–16).

Overexpression of GDF15 shifts the TME toward an immunosuppressive, tumor-promoting state by decreasing dendritic cell (DC) activation, reducing cytotoxic CD8^+^ T-cell infiltration, and stimulating CD4^+^ regulatory T-cell expansion (4, 7, 17–20). *In vitro* studies have shown that neutralizing GDF15 increases T-cell proliferation, promotes T-cell infiltration, and leads to the activation of DCs (17, 21).

GDF15 inhibition is associated with weight gain, and GDF15 overexpression is believed to mediate cancer-associated anorexia/cachexia by activating GFRAL (3, 10, 15, 22–24). GDF15 suppression has been shown to be beneficial in murine models of cancer cachexia (23, 25). In a phase II study of patients with advanced cancer, elevated serum GDF15, and cachexia, the GDF15 inhibitor ponsegromab was associated with a median weight gain of approximately 2.81 kg compared with placebo at 12 weeks (24).

AZD8853 is an IgG1 mAb that specifically binds to and neutralizes GDF15. In preclinical studies, AZD8853 restored differentiation and activation of human monocyte-derived DCs and T cells *in vitro* and led to substantial tumor regression associated with enhanced immune cell infiltration in the TME in mice harboring anti–PD-L1–refractory tumors (5). Here, we report results from the first-in-human phase I/IIa study of AZD8853 monotherapy in patients with previously treated advanced/metastatic solid tumors. Given the overexpression of GDF15 in colorectal cancer, NSCLC, and urothelial carcinoma, along with the prognostic association and high unmet clinical need, these tumor types were selected for the initial clinical study.

## Materials and Methods

### Study design

This was a first-in-human, phase I/IIa, open-label, multicenter study evaluating AZD8853 in patients with selected advanced/metastatic solid tumors (NCT05397171). The overall study design included substudy 1 to evaluate AZD8853 monotherapy in dose-escalation and dose-expansion phases. No additional substudies were added as the study was terminated after the dose-escalation phase of substudy 1.

The primary objective was to evaluate the safety and tolerability of AZD8853. Secondary objectives included preliminary efficacy, pharmacokinetics (PK), and pharmacodynamics (PD). Exploratory objectives included biomarkers of clinical activity and effects on cancer cachexia.

### Patients

Eligible patients were of ≥18 years of age with histologically or cytologically confirmed diagnosis of locally advanced or metastatic NSCLC, urothelial carcinoma, or microsatellite-stable colorectal cancer (MSS-CRC). Patients must have had documented progression on previous standard-of-care therapy for their tumor type: at least one prior line of systemic therapy for advanced/metastatic disease containing an anti–PD-(L)1 agent with or without platinum-based chemotherapy for NSCLC; at least two prior lines of systemic therapy for advanced/metastatic disease, including fluoropyrimidines, irinotecan, and oxaliplatin (unless refused or contraindicated) for MSS-CRC; or at least one prior line of systemic therapy, including a platinum-based regimen and/or anti–PD-(L)1 agent for urothelial carcinoma. Key exclusion criteria included prior therapy targeting GDF15 or GFRAL, symptomatic central nervous system metastases or leptomeningeal disease, unresolved grade ≥2 toxicities (NCI Common Terminology Criteria for Adverse Events version 5.0]) from prior therapy, active interstitial lung disease, active or prior autoimmune or inflammatory disorders, or type 2 diabetes managed by metformin. If previously exposed to immunotherapy targeting PD-(L)1 or CTLA-4, patients must not have experienced toxicity resulting in discontinuation, any adverse events (AE) that required the use of immunosuppression other than corticosteroids, a neurologic or ocular AE of any grade, or an immune-mediated AE of grade ≥3.

### Treatment

Based on preclinical toxicity, PK, and modeled human exposure, AZD8853 monotherapy doses of 300, 1,000, and 3,000 mg were selected for the dose-escalation phase. AZD8853 was given intravenously every 3 weeks until disease progression, unacceptable toxicity, or withdrawal of consent (Supplementary Fig. S1).

### Endpoints and assessments

Primary endpoints included characterizing AEs, serious AEs (SAE), AEs leading to discontinuation of AZD8853, and dose-limiting toxicities (DLT); determining the MTD of AZD8853; and establishing the recommended phase II dose of AZD8853. AEs were graded according to the NCI Common Terminology Criteria for Adverse Events version 5.0 and coded using the Medical Dictionary for Regulatory Activities version 26.0. Key secondary endpoints included objective response (OR) according to RECIST version 1.1, change in target lesion size from baseline, change in ctDNA levels from baseline, and free serum GDF15 levels. Exploratory endpoints included changes in peripheral immune cells and body weight.

### Statistical analyses

Dose escalation followed a modified toxicity probability interval 2 design and was based on the DLT evaluable set, defined as enrolled patients who completed safety assessments in the DLT evaluation period (21 days after receiving the first AZD8853 infusion) with at least 75% dosing, or who experienced any DLT during the DLT evaluation period. The safety and tolerability endpoints were summarized using descriptive statistics for the safety set, defined as all patients who received any amount of AZD8853. Efficacy analysis, PK, and PD also used descriptive statistics. Sample size for dose escalation was planned as three to nine evaluable patients per dose cohort.

### Trial oversight

The study was conducted in accordance with the Declaration of Helsinki, the Council for International Organizations of Medical Sciences International Ethical Guidelines, and all applicable International Conference on Harmonization Good Clinical Practice guidelines and laws and regulations. The protocol and all modifications were approved by relevant ethics committees and regulatory authorities. All patients provided written informed consent.

### Data availability

The data described in this article can be requested through Vivli at https://vivli.org/members/enquiries-about-studies-not-listed-on-the-vivli-platform. To ensure that the data are used for valid scientific purposes and to benefit patients, all requests are subjected to review and approval in accordance with AstraZeneca’s data sharing policy (https://www.astrazenecaclinicaltrials.com/our-transparency-commitments).

## Results

### Patients

Sixteen patients were treated across three dose levels: 300 mg (*n* = 3), 1,000 mg (*n* = 6), and 3,000 mg (*n* = 7). Fifteen patients had MSS-CRC, and one patient had urothelial carcinoma of the upper urinary tract; none had NSCLC. Overall, patients were representative of the intended population of patients with cancer (Supplementary Table S1). The median age was 62.5 years, 87.5% had an Eastern Cooperative Oncology Group performance status of 1, and the median number of prior systemic therapies was 4 (range, 2–9; Table 1). By June 6, 2023, all patients had discontinued AZD8853 treatment, and the study had been terminated. The most common reason for treatment discontinuation was objective progressive disease (14/16, 87.5%); one patient discontinued because of subjective disease progression, and one discontinued because of an AE (grade 5 cardiac arrest, not deemed to be treatment related). The median duration of exposure to AZD8853 was 44 days (range, 22–115) with a median of two cycles received (range, 1–5).

**Table 1:** Patient demographics and disease characteristics

Characteristic	AZD8853	AZD8853	AZD8853	Total
	300 mg	1,000 mg	3,000 mg	
	*n* = 3	*n* = 6	*n* = 7	*N* = 16
Median age (range), years	57.0 (52–77)	62.5 (58–69)	67.0 (53–75)	62.5 (52–77)
Male/female, *n* (%)	1 (33.3)/2 (66.7)	4 (66.7)/2 (33.3)	4 (57.1)/3 (42.9)	9 (56.3)/7 (43.8)
Race, *n* (%)				
Asian/White/not reported	0/3 (100)/0	0/5 (83.3)/1 (16.7)	1 (14.3)/5 (71.4)/1 (14.3)	1 (6.3)/13 (81.3)/2 (12.5)
ECOG performance status 0/1, *n* (%)	0/3 (100)	1 (16.7)/5 (83.3)	1 (14.3)/6 (85.7)	2 (12.5)/14 (87.5)
Primary tumor location, *n* (%)				
Colon	3 (100)	4 (66.7)	5 (71.4)	12 (75.0)
Rectum	0	1 (16.7)	2 (28.6)	3 (18.8)
Ureter	0	1 (16.7)	0	1 (6.3)
Disease stage at diagnosis^a^, *n* (%)				
II/III/IV/missing	0/1 (33.3)/2 (66.7)/0	0/2 (33.3)/4 (66.7)/0	1 (14.3)/3 (42.9)/2 (28.6)/1 (14.3)	1 (6.3)/6 (37.5)/8 (50.0)/1 (6.3)
Metastatic sites^b^, *n* (%)	3 (100)	6 (100)	7 (100)	16 (100)
Lung	3 (100)	4 (66.7)	6 (85.7)	13 (81.3)
Liver	3 (100)	2 (33.3)	4 (57.1)	9 (56.3)
Peritoneum/omentum	0	1 (16.7)	1 (14.3)	2 (12.5)
Bone	1 (33.3)	1 (16.7)	0	2 (12.5)
Other	1 (33.3)	1 (16.7)	5 (71.4)	7 (43.8)
Any surgical history on primary tumor, *n* (%)	1 (33.3)	6 (100)	6 (85.7)	13 (81.3)
Prior lines of anticancer therapy, median (range)	3.0 (2–9)	4.5 (4–6)	3.0 (2–7)	4.0 (2–9)
Prior anti-EGFR therapy, *n* (%)	1 (33.3)	4 (66.7)	1 (14.3)	6 (37.5)

a Based on American Joint Committee on Cancer, Cancer Staging Systems.

b Patients may have more than one location of metastatic site indicated; numbers in the groups may not add up to *n*.

Abbreviations: ECOG, Eastern Cooperative Oncology Group; EGFR, epidermal growth factor receptor.

### Safety

AZD8853 was well tolerated at all dose levels, and no safety concerns were identified (Table 2). Of the 16 patients, 13 (81.3%) had treatment-emergent AEs; the most common were diarrhea (31.3%), abdominal pain (31.3%), and decreased appetite (25.0%; Table 3). Eight (50.0%) patients experienced grade ≥3 treatment-emergent AEs, and six (37.5%) had SAEs, none related to AZD8853 (Table 2). Treatment-related AEs (TRAE) occurred in three (18.8%) patients: one had decreased appetite and diarrhea, both grade 1; one had grade 1 muscular weakness; and one had grade 2 weight increased; none of these events led to treatment discontinuation. There were no DLTs. As described above, one patient died because of an SAE of cardiac arrest, unrelated to AZD8853; the patient also had an SAE of pulmonary embolism unrelated to AZD8853 and a history of coronary artery disease and coronary stenting. Two patients had AEs leading to dose interruption, none considered treatment related, and no patient had an AE leading to dose modification.

**Table 2:** Safety summary

*n* (%)	AZD8853	AZD8853	AZD8853	Total
	300 mg	1,000 mg	3,000 mg	
	*n* = 3	*n* = 6	*n* = 7	*N* = 16
Any AEs	3 (100)	4 (66.7)	6 (85.7)	13 (81.3)
Possibly study treatment related	1 (33.3)	0	2 (28.6)	3 (18.8)
Grade ≥3 AEs	1 (33.3)	3 (50.0)	4 (57.1)	8 (50.0)
Possibly study treatment related	0	0	0	0
SAEs	1 (33.3)	3 (50.0)	2 (28.6)	6 (37.5)
Possibly study treatment related	0	0	0	0
SAEs with an outcome of death	0	1 (16.7)^a^	0	1 (6.3)
Possibly study treatment related	0	0	0	0
AEs leading to discontinuation of AZD8853	0	1 (16.7)^a^	0	1 (6.3)
Possibly study treatment related	0	0	0	0
AEs leading to interruption of AZD8853	0	1 (16.7)	1 (14.3)	2 (12.5)
SAEs leading to discontinuation of AZD8853	0	1 (16.7)^a^	0	1 (6.3)
Possibly study treatment related	0	0	0	0

a Death due to SAE of cardiac arrest, unrelated to AZD8853; this patient also had an SAE of pulmonary embolism, unrelated to AZD8853, and a history of coronary artery disease and coronary stenting.

**Table 3:** Treatment-emergent AEs occurring in >10% of total patient population at any grade

Safety set, *n* (%)	AZD8853, 300 mg *n* = 3		AZD8853, 1,000 mg *n* = 6		AZD8853, 3,000 mg *n* = 7		Total *N* = 16	
	Any grade	Grade ≥3	Any grade	Grade ≥3	Any grade	Grade ≥3	Any grade	Grade ≥3
Abdominal pain	1 (33.3)	0	1 (16.7)	0	3 (42.9)	2 (28.6)	5 (31.3)	2 (12.5)
Diarrhea	1 (33.3)	1 (33.3)	1 (16.7)	0	3 (42.9)	0	5 (31.3)	1 (6.3)
Decreased appetite	2 (66.7)	0	0	0	2 (28.6)	1 (14.3)	4 (25.0)	1 (6.3)
Constipation	1 (33.3)	0	1 (16.7)	0	1 (14.3)	0	3 (18.8)	0
Cough	1 (33.3)	0	1 (16.7)	0	1 (14.3)	0	3 (18.8)	0
Fatigue	0	0	1 (16.7)	0	2 (28.6)	1 (14.3)	3 (18.8)	1 (6.3)
Pyrexia	2 (66.7)	0	0	0	1 (14.3)	0	3 (18.8)	0
Back pain	0	0	2 (33.3)	1 (16.7)	0	0	2 (12.5)	1 (6.3)
Chills	1 (33.3)	0	0	0	1 (14.3)	0	2 (12.5)	0
Insomnia	0	0	1 (16.7)	0	1 (14.3)	0	2 (12.5)	0

### Efficacy

**Figure 1:**
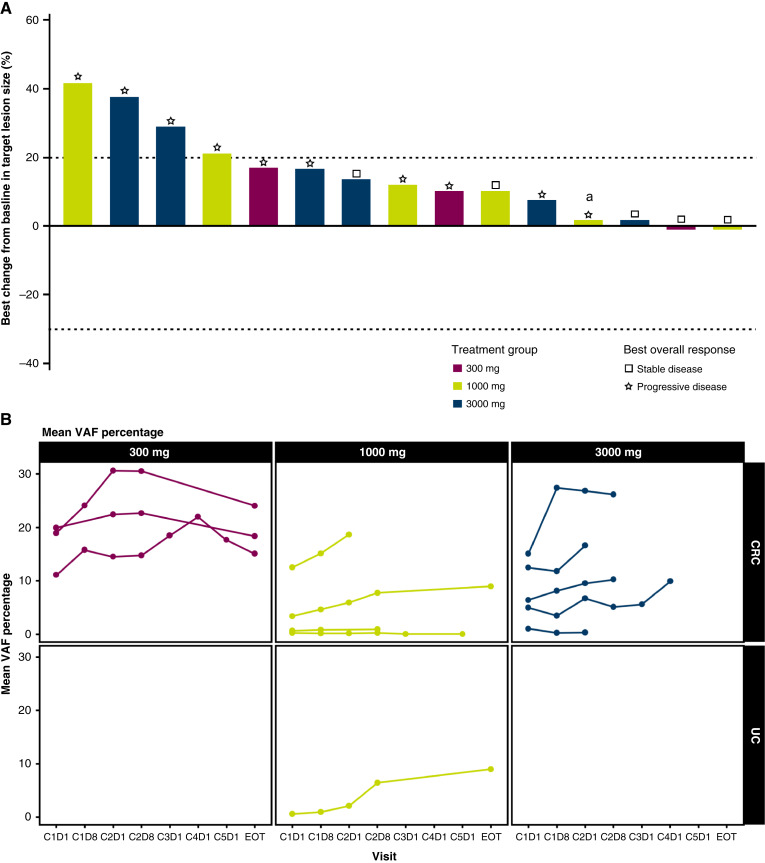
Efficacy of AZD8853 in patients with previously treated advanced/metastatic solid tumors assessed by best change from baseline in target lesion size (**A**) and ctDNA clearance (**B**). Antitumor response was measured by best percentage change in target lesion size from baseline using standard radiographic imaging. Best overall response was based on investigator assessment according to RECIST v1.1 (**A**). Clearance of ctDNA was measured from whole blood samples (**B**). ^a^Patient had UC; all other patients had CRC. C, cycle; CRC, colorectal cancer; D, day; EOT, end of treatment; UC, urothelial carcinoma; VAF, variant allele frequency.

There were no ORs according to RECIST v.1.1. Five (31.3%) patients had stable disease ≥5 weeks as their best response, and 11 (68.8%) patients had disease progression as the best response (Fig. 1A). Of those 11 patients, 10 had radiological progressive disease, and one died because of progressive disease prior to the first follow-up assessment. There were no consistent reductions in target lesion size (Fig. 1A) or any evidence of ctDNA clearance irrespective of dose (Fig. 1B).

One patient had grade 3 weight decreased and an SAE of small intestinal obstruction (both unrelated to AZD8853), and one patient had grade 2 weight increased possibly related to AZD8853. Both patients were in the 3,000 mg dosing cohort. There were no grade 1 body weight AEs and no apparent overall trends in body weight change during treatment (Supplementary Fig. S2).

### PK and PD

**Figure 2:**
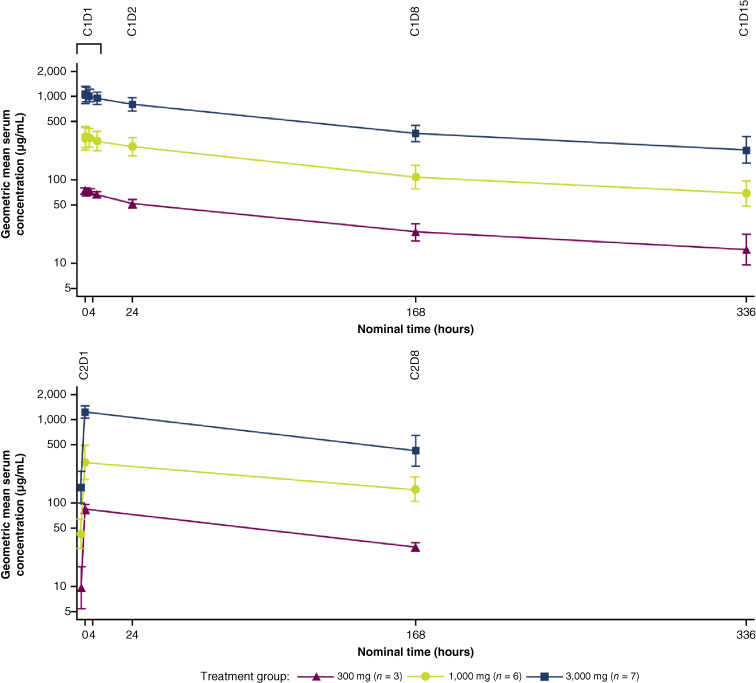
Geometric mean (gSD) serum concentrations (μg/mL) of AZD8853 vs. time (semilogarithmic scale) – PK set. Vertical lines represent the geometric mean ± gSD. Geometric mean − gSD: exp (mean[log(PK Conc)] − std [log(PK Conc)]). Geometric mean + gSD: exp (mean[log(PK Conc)] + std [log(PK Conc)]). C, cycle; D, day; gSD, geometric SD; PK Conc, PK concentration.

**Figure 3:**
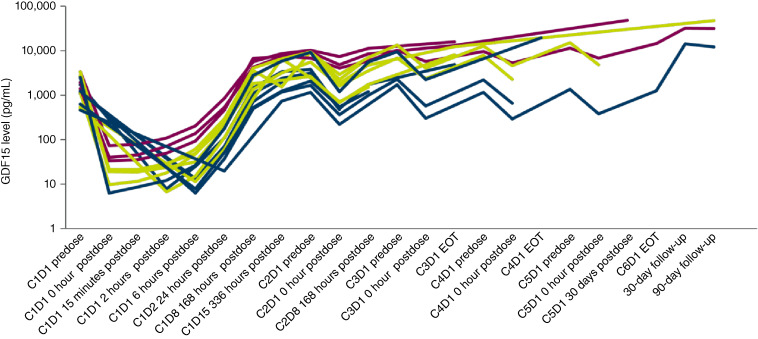
Free serum GDF15 following AZD8853 treatment. Whole blood samples were collected at various timepoints for evaluation of free serum GDF15. C, cycle; D, day; EOT, end of treatment.

AZD8853 had linear PK within the dose range tested, with a half-life of ∼5 to 10 days and dose-proportional increases in exposure, supporting every 3 weeks dosing (Table 4; Fig. 2). Serum concentrations of free GDF15 initially decreased following intravenous administration of AZD8853, with a reduction of >99% postdose at all dose levels. However, GDF15 concentrations returned to and exceeded predose concentrations at 7 days after dose (Fig. 3). The magnitude of the observed rebound was reduced in a dose-dependent manner, with the 300 mg dose resulting in cycle 2 predose serum GDF15 concentrations being ∼6-fold higher than baseline concentrations, but only 5-fold higher for the 1,000 mg dose and 3-fold higher for the 3,000 mg dose.

**Table 4:** AZD8853 PK parameters [geometric mean (geometric CV %)] – PK set

AZD8853 dose	*N*	Cmax, μg/mL	AUC_(t1–t2)_, hours*μg/mL	AUC_last_, hours*μg/mL	AUC_inf_, hours*μg/mL	t_1/2_, hours^a^	Cl, L/hours	Vz, L
300 mg	3	76.65 (4.515)	11,700 (21.24)	9,979 (17.88)	14,140 (32.32)	208.4 (113–242)	0.02122 (32.32)	5.474 (13.25)
1,000 mg	6	328.2 (29.21)	55,580 (27.38)	47,900 (26.34)	65,260 (29.36)	172.4 (132–216)	0.01532 (29.36)	3.719 (25.10)
3,000 mg	7	1,103 (19.32)	183,200 (27.45)	156,800 (24.77)	213,300 (32.96)	155.6 (140–240)	0.01406 (32.96)	3.362 (17.95)

a Data for this parameter are presented for median and range (minimum and maximum).

Abbreviations: Cl, total body clearance; Cmax, maximum concentration; CV, coefficient of variation; t_1/2_, half-life; Vz, volume of distribution in the terminal phase.

**Figure 4:**
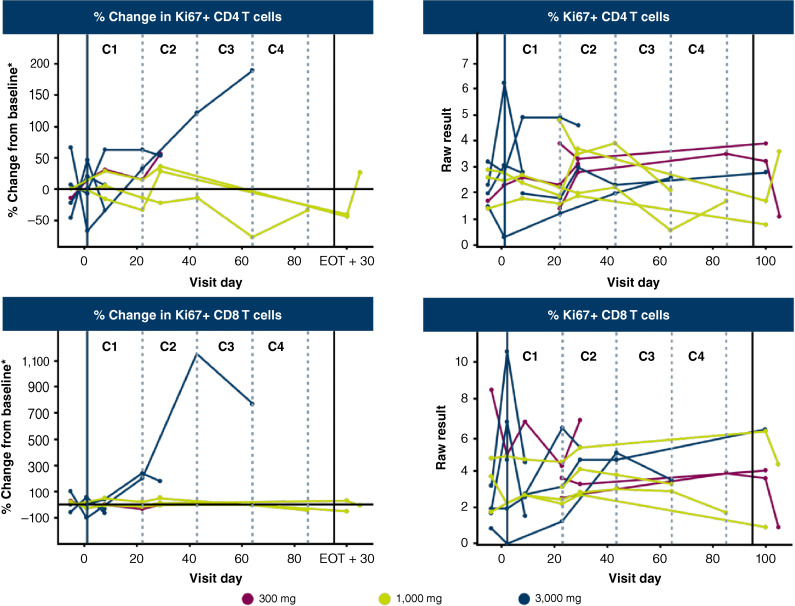
Changes in peripheral T cells during AZD8853 treatment. The patient with a strong increase in proliferating T cells (left plots) started with a very low number at baseline (right plots). Whole blood samples were collected at various timepoints for analysis of numbers of peripheral T cells using flow cytometry–based immunophenotyping of circulating lymphocytes. *Baseline is the average of the screening and C1D1 predose (visit day 0). C, cycle; D, day; EOT, end of treatment; Ki67+, antigen Kiel 67 positive.

There was no evidence of consistent changes in peripheral immune cells, including proliferating (Ki67+) CD8^+^ and CD4^+^ T cells (Fig. 4). The trial was terminated early because of insufficient sustained decreases of free serum GDF15 at the doses evaluated and the lack of clinical activity based on PD and efficacy objectives. The MTD was not reached, and the recommended phase II dose was not determined.

## Discussion

In this first-in-human study, AZD8853 monotherapy was well tolerated in patients with MSS-CRC and urothelial carcinoma. Three patients had TRAEs, which were of low grade and did not lead to treatment discontinuation. The highest severity TRAE was grade 2 weight increased in one patient. This may have been a consequence of ascites accumulation or a pharmacologic effect of AZD8853, as GDF15 is known to affect metabolic activity, although no consistent effects on body weight were seen in the overall patient population. There were no DLTs at any dose level.

AZD8853 did not have measurable efficacy at the three dose levels tested, as shown by RECIST assessments. There was no substantial decline in ctDNA across all dose levels. The absence of clinical benefit was possibly related to the transient reduction of free serum GDF15. The study was terminated, and no further development of AZD8853 is planned.

There are several potential explanations for the apparent rebound of GDF15 over baseline concentrations. Target turnover is a key factor in the suppression of any soluble target; fast turnover may limit the suppressive potential of a therapy because of a lack of engagement time prior to target washout. Slow turnover leads to accumulation of drug:target complexes that act as a target reservoir; as drug:target complexes accumulate, they can shift the dynamic equilibrium of the drug:target interaction toward dissociation and release of free target (26). GDF15 may have been present in tissue reservoirs and released into circulation when the soluble GDF15 was suppressed by AZD8853 (26).

The apparent return to baseline and subsequent rebound could also be driven by bioanalytic assay considerations. Antibody:target interactions lead to multiple species of analytes *in vivo*, including free antibody, free target, and antibody:target complex (27). In the case of dimeric targets such as GDF15, the range of potential complexes is greater because of the presence of a greater number of epitopes for antibody binding, which could have presented analytic challenges for free-ligand binding assay used in this study. It is possible that the bioanalytic process disrupted the dynamic equilibrium of the drug:target complex and increased the apparent concentration of the free target. An assay quantifying total target, including free and bound GDF15 in all of its potential configurations might have yielded different results.

Single-agent AZD8853 did not lead to an increase in Ki67+ CD4^+^ and CD8^+^ T cells in peripheral circulation, whereas this effect is often observed with other types of immuno-oncology agents such as PD-(L)1 inhibitors (28–30). This could reflect, as noted above, the failure to sustain free GDF15 suppression, or it might suggest the presence of other more potent immunosuppressive factors beyond GDF15. In addition, the effects of GDF15 neutralization could be more apparent in the TME; for example, the anti-GDF15 antibody visugromab (formerly CTL-002) was reported to increase intratumoral T-cell infiltration and proliferation in the phase I portion of the GDFATHER trial (31). However, paired tumor biopsies were not available for the current study.

The lack of clear trend in body weight change during AZD8853 treatment could reflect an absence of sustained effects on GDF15. Elevated GDF15 concentrations are associated with weight loss and poor outcomes in patients with various types of cancer (15). The anti-GDF15 antibody ponsegromab suppressed serum unbound GDF15 concentrations and was associated with weight gain in patients with advanced cancer in a phase Ib study and led to weight gain and improved cancer cachexia symptoms in patients with cancer in a phase II study (24, 32).

Although other studies have investigated the effects of GDF15-neutralizing antibodies in the treatment of cancers, this was the first study to explore the antitumor activity of AZD8853 monotherapy. Preliminary results from the phase IIa portion of the ongoing GDFATHER study showed that combining the anti-GDF15 antibody visugromab with the PD-1 inhibitor nivolumab resulted in an OR rate of 16.7% across patients with advanced NSCLC, urothelial carcinoma, or hepatocellular carcinoma who had relapsed on or were refractory to immune checkpoint inhibitors (20, 33, 34). This is slightly higher than what has been reported in retrospective studies of immune checkpoint inhibitor rechallenge after progressive disease in nonmelanoma cancers (35), but controlled trials will be necessary to confirm whether GDF15 neutralization can restore anti–PD-1 activity in previously treated patients.

The study limitations include incomplete characterization of free, partially bound, and totally bound serum GDF15 levels, as well as the inability to quantify GDF15 in the TME due to the lack of paired biopsy samples. Tumor biopsies could also have provided insights into changes in the immune cell population in the TME. The study enrolled predominantly patients with MSS-CRC, who are known to be nonresponsive to existing immunotherapies, and only one patient with a tumor type other than MSS-CRC. The study employed a 3-week dosing regimen based on the half-life of AZD8853 but may have benefitted from alternative dosing schedules or higher-dose regimens to assess whether GDF15 suppression would be sustained. However, a substantially higher dose would not have been feasible for practical reasons including cost of goods, production limits, and potential concerns about safety and duration of administration. Additionally, preliminary PK–PD modeling did not indicate that an every 2 weeks dosing schedule would achieve sustained target suppression. Combinations of AZD8853 with other immunotherapies were also not explored. Further studies on the effects of GDF15 suppression with AZD8853 were not conducted because of the transient nature of the suppression.

In conclusion, AZD8853 had a favorable safety profile in patients with advanced solid tumors, but did not demonstrate antitumor activity or sustained reduction of free GDF15.

## Supplementary Material

Supplementary Data Supplementary Materials and Methods, Supplementary Table S1

Figure S1 Study design

Figure S2 Body weight change from baseline during AZD8853 treatment.
