# Supplementary material for: First-in-Human Study to Evaluate the Safety and Efficacy of Anti-GDF15 Antibody AZD8853 in Patients with Advanced/Metastatic Solid Tumors
Source: Cancer Res Commun. 2025 Jun 2;5(6):896–905. doi: 10.1158/2767-9764.CRC-24-0565 (PMC12127903; doi:10.1158/2767-9764.CRC-24-0565)
Supplement: Supplementary Data — Supplementary Materials and Methods, Supplementary Table S1 [file crc-24-0565_supplementary_data_suppsmm.docx]

**SUPPLEMENTARY MATERIALS**

**SUPPLEMENTARY MATERIALS AND METHODS**

**ctDNA**

Baseline plasma ctDNA samples were sequenced using the GuardantOMNI assay and longitudinal samples were profiled with the Guardant360 CDx assay. Mean VAF (variant allele frequency) was calculated across all patients for whom data was available and was plotted for colorectal cancer (CRC) and urothelial cancer (UC) at the following timepoints: C1D1, C1D8, C2D1, C2D8, C3D1, C4D1, C5D1 and end of treatment.

**Flow cytometry**

*TBNK cell assay*

Blood collected in tubes containing sodium heparin as an anticoagulant was used for the analysis of T cells, B cells, and NK cells. Whole blood (50 μl) was added to a tube containing 18.75 μl of antibody cocktail. This cocktail included the following fluorochrome-conjugated antibodies: CD19 BV421 (clone HIB19, Cat#562440, RRID: AB_11153299), CD4 BV510 (clone SK3, Cat#562971, RRID: AB_2744424), CD3 FITC (clone SK7, Cat#345764, RRID: AB_2916364), CD56 PE (clone NCAM16.2, Cat#345812, RRID: AB_2629216), CD16 PE (clone B73.1, Cat#332779, RRID: AB_2868628), CD45 PerCPCy5.5 (clone 2D1, Cat#332784, RRID: AB_2868632), CD8 APC (clone SK1, Cat#345775, RRID: AB_2868803) and CD14 APC-H7 (clone MφP9, Cat#641394, RRID: AB_1645725); all from BD Biosciences. The tube was briefly vortexed and incubated at room temperature (RT) for 15 minutes protected from light. Subsequently, erythrocytes were lysed by the addition of 450 μl of 1x BD FACS™ Lyse solution (BD Biosciences) and incubated at RT protected from light for an additional 15 minutes. Tube contents were then analyzed using a BD FACSCanto™ II flow cytometer (RRID: SCR_018056) running BD FACS™ Diva (RRID: SCR_001456). Helper and cytotoxic T cell subsets were identified as cells expressing CD4 or CD8, respectively, within the CD3+ population. Both B cells and NK cells were identified from the non-CD3 expressing population with the former being positive for CD19 and the latter being positive for CD16 and/or CD56. Monocytes were identified within the leukocyte population by CD14 expression. Absolute counts were calculated using a dual platform method. The total leukocyte count was calculated by combining the number of events in the lymphocyte gate (SSC vs CD45 PerCP-Cy5.5) with the number of events in the monocyte gate (SSC vs CD14 APC-H7). The lymphocyte:leukocyte ratio was calculated by dividing the number of events in the lymphocyte gate (CD3 BV421 vs CD45 PerCP-Cy5.5) by the total leukocyte count. The lymphocyte:leukocyte ratio was multiplied by the white blood cell count from a hematology analyzer. CD14+ monocytes were reported as relative percentages of the total leukocyte count directly from the hematology analyzer.

*Activation and Proliferation of TNK Cell Subsets Assay*

Whole blood collected in sodium heparin tubes was used for the analysis of T cell proliferation. A volume of 50 μl of whole blood was added to a tube containing 18.75 μl of a premixed antibody cocktail. This cocktail included the following fluorochrome-conjugated antibodies: CD4 BUV396 (clone SK3, BD Biosciences Cat#563550, RRID: AB_2738273), CD16 BUV496 (clone 3G8, BD Biosciences Cat#612944, RRID: AB_2870224), CD25 BUV615 (clone M-A251, BD Biosciences Cat#751331, RRID: AB_2875340), CD38 BUV661 (clone HB7, BD Biosciences Cat#612969, RRID: AB_2870242), CD69 BUV737 (clone FN50, BD Biosciences, Cat#612817, RRID: AB_2870141), CD56 BUV805 (clone NCAM16.2, BD Biosciences Cat#749086, RRID: AB_2873478), TCR γδ BV421 (clone 11F2, BioLegend Cat#331218, RRID: AB_2562317), CD11b SB436 (clone ICRF44, Thermo Fisher Cat#62-0118-42, RRID: AB_2662533), CD45RA eFlour 450 (clone HI100, Thermo Fisher Cat#48-0458-42, RRID: AB_1272059), Ki-67 BV480 (clone B56, BD Biosciences Cat#566109, RRID: AB_2739511), CD57 BV510 (clone NK-1, BioLegend Cat#393314, RRID: AB_2750342), CD3 BV570 (clone SK7, BioLegend Cat#300436, RRID: AB_2562124), CD95 BV605 (clone DX2, BioLegend Cat#305628, RRID: AB_2563825), CD279 BV650 (clone EH12.1, BD Biosciences Cat#564104, RRID: AB_2738595), HLA-DR BV750 (clone G46-6, BioLegend Cat#307672, RRID: AB_2800802), CD197 BV785 (clone 3D12, BioLegend Cat#353230, RRID: AB_2563630), CD96 BB515 (clone TH111, BD Biosciences Cat#564774, RRID: AB_2738945), CD19 Alexa 488 (clone HIB19, BD Biosciences Cat#557697, RRID: AB_2649928), FOXP3 PE (clone PCH101, BioLegend Cat#320207, RRID: AB_492983), Granzyme B PE-Dazzle 594 (clone GB11, BioLegend Cat#372216, RRID: AB_2728383), CD27 PerCP-Cy5.5 (clone M-T271, BioLegend Cat#393210, RRID: AB_2750095), CD14 PerCP-eFluor 710 (clone M5E2, Thermo Fisher Cat#46-0149-42, RRID: AB_10671405), CD122 PE-Cy7 (clone CF1, Beckman Coulter Cat#A53365), TIGIT APC (clone A15153G, R&D Systems Cat#FAB7898A, RRID: AB_3652762), CD314 Alexa 660 (clone 1D11, custom conjugation), CD8 APC-H7 (clone SK1, BD Biosciences Cat#641400, RRID: AB_1645736). The tube was processed and erythrocytes lysed as described above. The tube contents were analyzed using the Cytek™ Aurora flow cytometer (RRID: SCR_019826) running SpectroFlo™ software (RRID: SCR_025494)*.* Proliferating T cell subsets (identified by Ki-67) were determined using the gating strategy specific to the assay. Assay was performance qualified by IQVIA (Durham, NC).

**SUPPLEMENTARY TABLE**

**Table S1.** Representativeness of study participants

| **Cancer type/subtype/stage/condition** | The study enrolled patients with histologically or cytologically confirmed locally advanced or metastatic urothelial carcinoma (UC, 6.3%) or microsatellite-stable colorectal cancer (MSS-CRC, 93.8%); considerations related to these two cancer types are further described below. |
| --- | --- |
| **Considerations related to:** | |
| **Sex** | Age-adjusted incidence rates for bladder cancer and CRC are higher for men (9.3 and 22.0 per 100,000, respectively) than for women (2.4 and 15.2 per 100,000, respectively).^1,2^ |
| **Age** | The median ages at diagnosis for bladder cancer and CRC are 73 and 66 years, respectively.^3,4^ |
| **Race/ethnicity** | In the US, White individuals are at the greatest risk of bladder cancer and American Indian/Alaska Native individuals are at the greatest risk of CRC compared to other racial and ethnic groups.^3,4^  Age-adjusted incidence for bladder cancer in males and females are 37.7 and 9.3 (White population), 20.0 and 5.1 (non-Hispanic American Indian/Alaska Native population), 18.9 and 5.8 (non-Hispanic Black population), 17.0 and 4.7 (Hispanic population) and 14.7 and 3.5 (non-Hispanic Asian/Pacific Islander population) per 100,000, respectively.^3^  Age-adjusted incidence for CRC in males and females are 53.6 and 45.5 (non-Hispanic American Indian/Alaska Native population), 49.4 and 36.8 (non-Hispanic Black population), 42.0 and 32.4 (White population), 39.4 and 28.7 (Hispanic population) and 34.5 and 25.3 (non-Hispanic Asian/Pacific Islander population) per 100,000, respectively.^4^ |
| **Geography** | Globally, the countries with the highest incidence of bladder cancer in 2022 were China, the US, and Italy; mortality was highest in China, the US, and India.^1^  Countries with the highest incidence of CRC in 2022 were China, the US, and Japan; mortality was also highest in these countries.^2^ |
| **Other considerations** | None |
| **Overall representativeness of this study** | The slightly uneven distribution of males (56.3%) and females (43.8%) in our study reflects the higher incidence of these cancers in males.  The age distribution of patients in our study  (median age 62.5 years [range 52–77]) is similar to the age distribution of these cancers.  Patients were enrolled in the US (81.3%) and Canada (18.8%), a region that has a high incidence of these cancers but is not representative of the global population of these cancers. |

References

1. World Cancer Research Fund International. Bladder cancer statistics. Available at: https://www.wcrf.org/preventing -cancer/cancer-statistics/bladder-cancer-statistics/. Accessed February 14, 2025.
2. World Cancer Research Fund International. Colorectal cancer statistics. Available at: https://www.wcrf.org/preventing -cancer/cancer-statistics/colorectal-cancer-statistics/. Accessed February 14, 2025.
3. National Cancer Institute. SEER cancer stat facts: bladder cancer. Available at http://seer.cancer.gov/statfacts/html/urinb.html. Accessed February 14, 2025.
4. National Cancer Institute. SEER cancer stat facts: colorectal cancer. Available at http://seer.cancer.gov/statfacts/html/colorect.html. Accessed February 14, 2025.
