# Supplementary material for: First-in-Human Study to Evaluate the Safety and Efficacy of Anti-GDF15 Antibody AZD8853 in Patients with Advanced/Metastatic Solid Tumors
Source: Cancer Res Commun. 2025 Jun 2;5(6):896–905. doi: 10.1158/2767-9764.CRC-24-0565 (PMC12127903; doi:10.1158/2767-9764.CRC-24-0565)
Supplement: Figure S1 — Study design [file crc-24-0565_figure_s1_suppsf1.pdf]

Figure S1. Study design

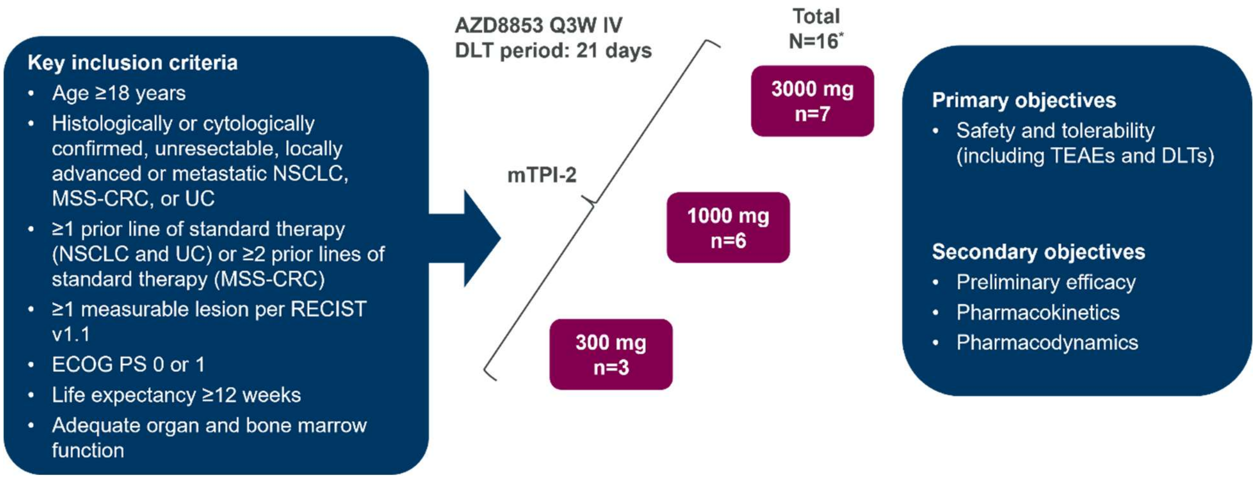

*Total number of patients enrolled; the study is now terminated

DLT, dose-limiting toxicity; ECOG PS, Eastern Cooperative Oncology Group performance status; IV, intravenous; MSS-CRC, microsatellite-stable colorectal cancer; mTPI-2, Modified Toxicity Probability Interval-2; NSCLC, non-small-cell lung cancer; Q3W, every 3 weeks; RECIST, Response Evaluation Criteria in Solid Tumors; TEAE, treatment-emergent adverse events; UC, urothelial carcinoma
