# Supplementary figures and images for: First-in-Human Study to Evaluate the Safety and Efficacy of Anti-GDF15 Antibody AZD8853 in Patients with Advanced/Metastatic Solid Tumors
Source: Cancer Res Commun. 2025 Jun 2;5(6):896–905. doi: 10.1158/2767-9764.CRC-24-0565 (PMC12127903; doi:10.1158/2767-9764.CRC-24-0565)

**Figure S2.** Body weight change from baseline during AZD8853 treatment.

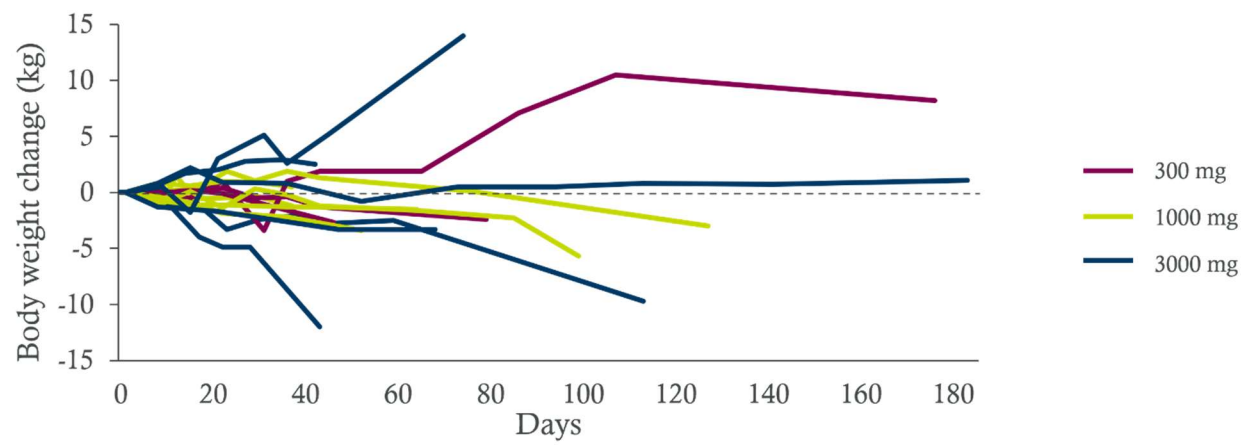

Supplement: Figure S2 — Body weight change from baseline during AZD8853 treatment. [file crc-24-0565_figure_s2_suppsf2.pdf]
